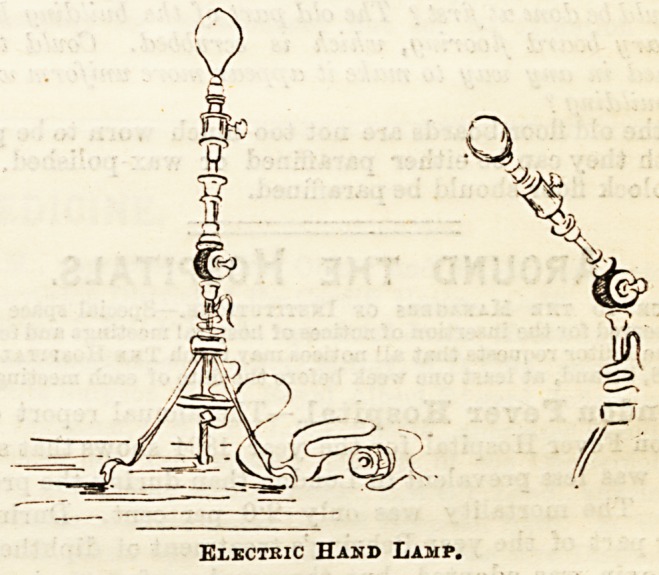# Ward Fittings at the London Temperance Hospital

**Published:** 1895-05-04

**Authors:** 


### THE INSTITUTIONAL WORKSHOP.

### PRACTICAL DEPARTMENTS.

### WARD FITTINGS AT THE LONDON TEMPERANCE HOSPITAL.

Few hospital wards in London can surpass those at the Temperance Hospital, Hampstead Road, for the completeness of all their fittings and appliances, and there is an especial atmosphere of comfort and well-being within them.

The use of glass has been often advocated as the best possible substance for the tops of lockers, doctors' tables, &c., and it is satisfactory to see that it is becoming very generally adopted for these purposes in those institutions which are most up to date in such matters. The glass-topped test tables at the Temperance Hospital are especially worthy of notice, one of which is illustrated below with the permission of Miss Orme, the matron, who has kindly allowed sketches of one or two of the more notable fittings in her wards to be made for reproduction here. Just inside the entrance to each ward are placed these slabs of dark-painted wood, covered with a thick sheet of plate glass, the general appearance being that of particularly well-kept black marble.

TEST TABLE.

Being quite impervious to the action of acids, it is, however, very superior in practical use to marble or tiles for ward purposes, and if it may be a little more trouble to clean, it certainly well repays the efforts spent upon it. Over the slab is fixed a bracket for holding testing tubes, and above that again hangs a neat little poison cupboard; the latter is not, however, included in our sketch.

An excellent dresser's table is shown in the second illustration. Moving easily and noiselessly on rubber wheels, it can be taken from bed to bed as required, and possesses ample space for everything needful in the way of dressings, bowls, and all the hundred and one things in demand in a hospital ward. The top shelf is of glass; the other three divisions are tiled. There are two drawers, and the lowest shelf is reserved for water cans, &c. It is altogether a very well-arranged piece of ward furniture.

The introduction of the electric light has been a great improvement to the appearance of the wards, and the comfort and convenience of patients, nurses, and doctors alike have been much increased thereby. Bell lights are attached to each bed, and a very useful and attractive-looking little apparatus is used as a hand lamp, of which we give an illustration. Standing on three supports, it is jointed so that light may be given at any angle required, and is further provided with a hook by which it may be securely hung upon the rail of the bedstead, or upon a ring in the wall, &c. These pleasant little hand lamps may be bought at quite moderate prices. These particular ones were procured, we believe, from Messrs. Shoolbred, Tottenham Court Road.

The light movable screens which have come into such general use of late years in hospital wards in place of the heavy and more or less clumsy articles of days gone by are

DRESSER'S TABLE.

now in many cases positive ornaments, and with their turkey red or cretonne covers help to give a bright and cheerful aspect to the wards of modern hospitals. Those at the Temperance Hospital will be objects of envy to visitors from less favoured institutions. They are particularly well made (from an American design), and are sufficiently strong to stand much wear, while at the same time they are light enough to be easily moved about the ward. The fourfold frame is of plain varnised wood, between which is stretched in rather full folds, cretonne of a dainty pattern and colour. Some carping critics of hospital economies are still to be found who

ELECTRIC HAND LAMP.

seem to look upon any such artistic developments as "out of place" in a charitable institution, and as necessarily meaning a waste of money; but it must be understood that very often the results which most please the eye are merely the consequence of a little trouble and forethought on the part of sister or matron, and mean the comparative difference, if any there be, of possibly a few pence in money, while that in appearance is everything. The moral, not to say physical, effect upon the occupants of hospital beds of evidences around them of cheerfulness and comfort as expressed in plenty of flowers, and in pictures and the introduction of things pleasant to the eye wherever possible, is not to be made light of, and fortunately is becoming better understood every day.

Patients at the Temperance Hospital have at any rate nothing to complain of in this respect, for it would be difficult to find wards of a more cheerful and pleasant aspect, and admiration and praise must be accorded to many of the appurtenances there to be found.

## Figures and Tables

**Figure f1:**
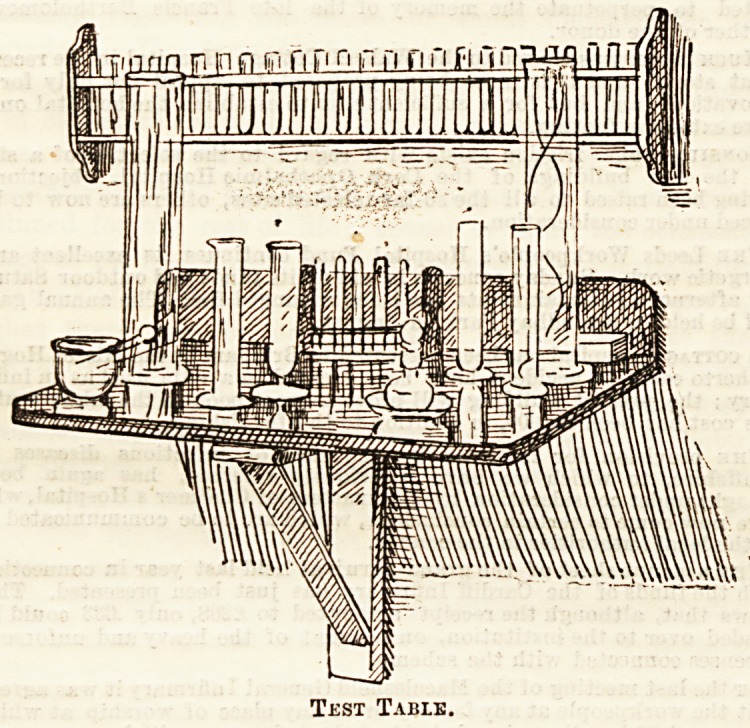


**Figure f2:**
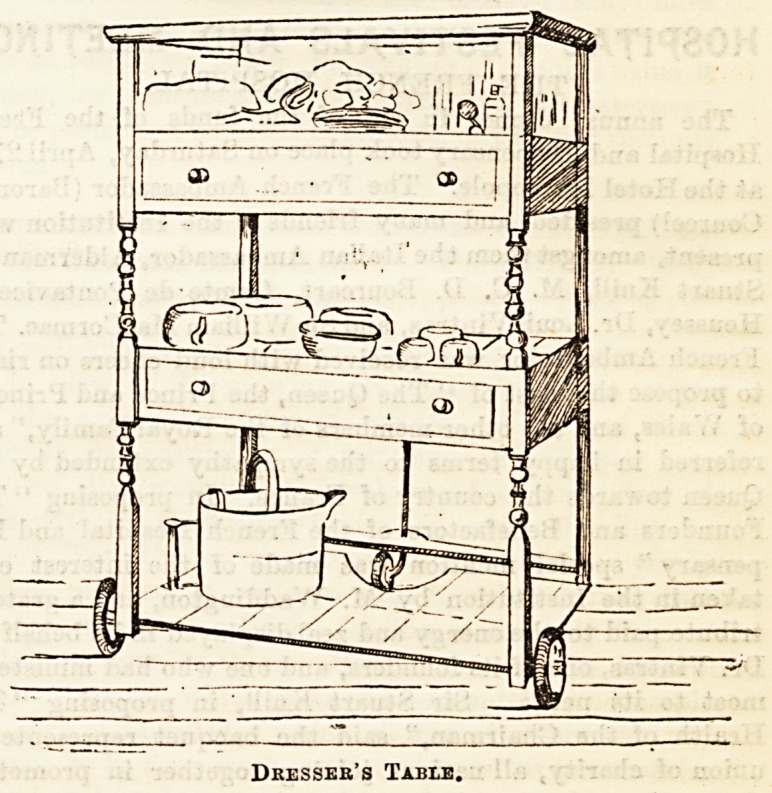


**Figure f3:**